# Supplementary material for: The role of 18F-FDG PET/CT in patients with serous cavity effusion of undetermined origin: a retrospective clinical study
Source: PeerJ. 2025 May 22;13:e19495. doi: 10.7717/peerj.19495 (PMC12103840; doi:10.7717/peerj.19495)
Supplement: Supplemental Information 2 [file peerj-13-19495-s002.doc]

**
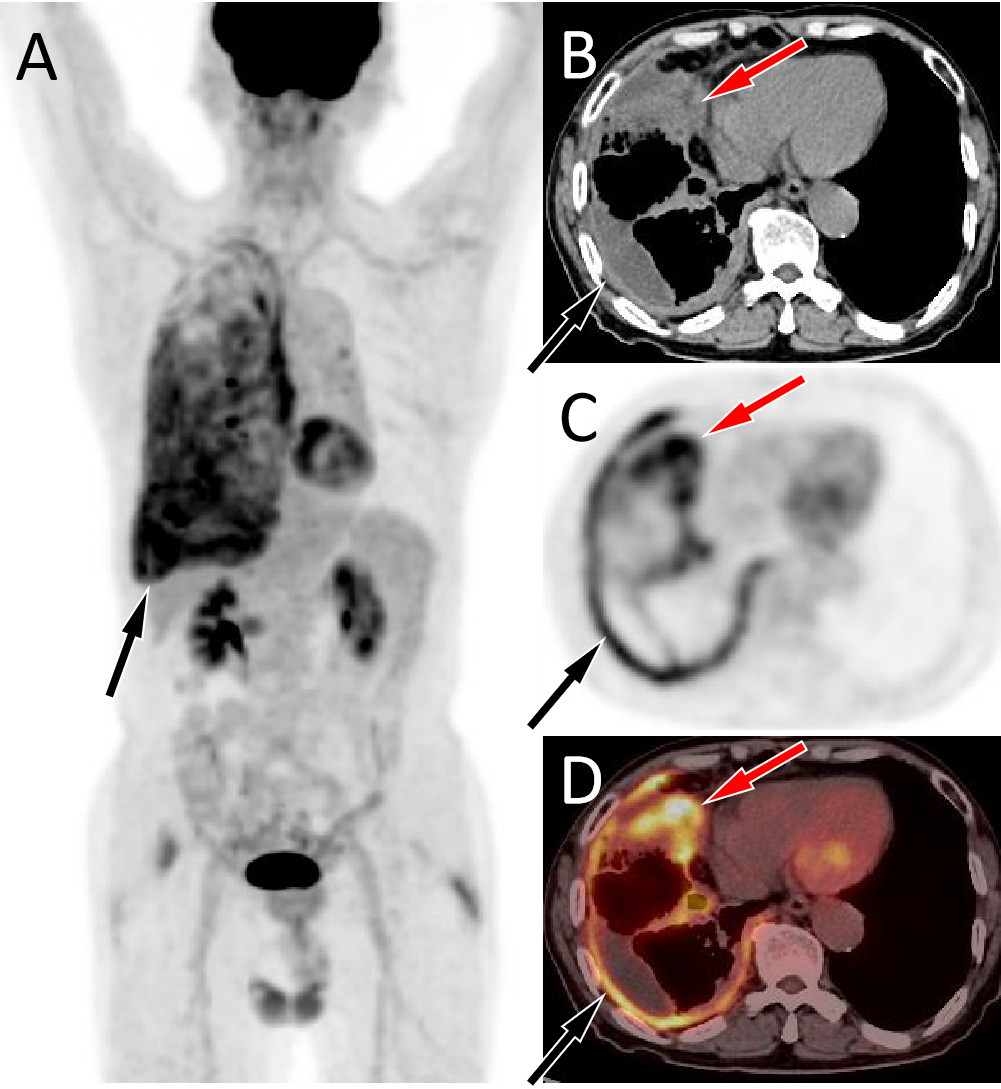
**

Fig. S1. PET/CT images of 78-y-old man who presented with pleural effusion. He was admitted to our hospital for further diagnosis and treatment after experiencing recurrent pleural effusion reaccumulation following thoracentesis performed at another hospital. The maximum intensity projection (MIP, **A**) revealed an increased ^18^F-FDG uptake in the right pulmonary field. Axial CT (**B**) showed a small amount of fluid in the right thoracic cavity (black arrow) and an uneven soft tissue density nodule in the middle lobe of the right lung (red arrow); axial PET (**C**) and axial fusion PET/CT (**D**) showed increased ^18^F-FDG uptake in right pleural effusion with a SUVmax of 6.0 (black arrows), and increased ^18^F-FDG uptake in right middle lobe nodule with a SUVmax of 7.5 (red arrows). Subsequently, needle biopsy confirmed adenocarcinoma in right middle lobe nodule, and adenocarcinoma cells were also identified in the right pleural fluid.

**
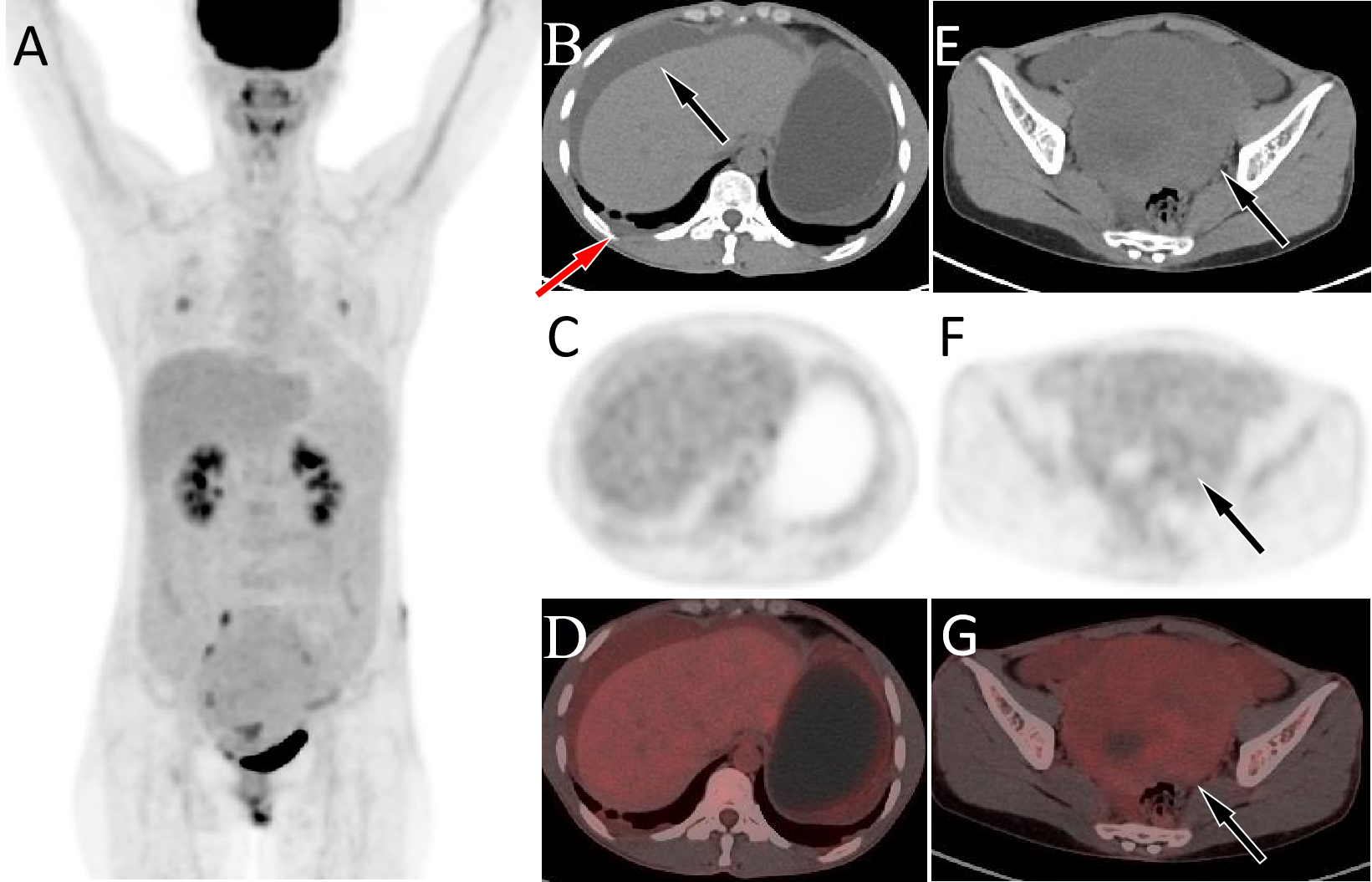
**

Fig.S2. PET/CT images of 39-y-old woman who presented with pelvic mass and ascites. The patient’s serum Ca125 was 269.0, and the levels of other serum tumor markers were within the normal range. The maximum intensity projection (MIP, **A**) showed that there was no significantly increased ^18^F-FDG uptake in the whole body scan. Axial CT (B) showed ascites and a small amount of fluid in the right pleural cavity, while no significant increased uptake of ^18^F-FDG was observed on PET (C) or PET/CT (D), with an SUVmax of 1.6. At the pelvic level, axial CT (**E**) showed an uneven soft tissue density mass (arrow), and there was also no increased ^18^F-FDG uptake on axial PET (**F**) and PET/CT (**G**), with a SUVmax of 2.1 (arrows), suggesting a benign tumor. The patient's postoperative pathological results confirmed the diagnosis of thecoma, which is consistent with our PET/CT findings.
